# Ultrasonic-assisted enzymatic improvement of polyphenol content, antioxidant potential, and *in vitro* inhibitory effect on digestive enzymes of Miang extracts

**DOI:** 10.1016/j.ultsonch.2023.106351

**Published:** 2023-03-01

**Authors:** Nalapat Leangnim, Kridsada Unban, Patcharapong Thangsunan, Suriya Tateing, Chartchai Khanongnuch, Apinun Kanpiengjai

**Affiliations:** a Program in Biotechnology, The Graduate School, Chiang Mai University, Chiang Mai 50200, Thailand; b Division of Biochemistry and Biochemical Innovation, Faculty of Science, Chiang Mai University, Chiang Mai 50200, Thailand; c Division of Food Science and Technology, Faculty of Agro-Industry, Chiang Mai University, Chiang Mai 50100, Thailand; d Research Center for Multidisciplinary Approaches to Miang, Chiang Mai University, Chiang Mai 50200, Thailand; e Center of Excellence in Fish Infectious Diseases (CE FID), Department of Veterinary Microbiology, Faculty of Veterinary Science, Chulalongkorn University, Bangkok 10330, Thailand; f Department of Plant and Soil Sciences, Faculty of Agriculture, Chiang Mai University, Chiang Mai 50200, Thailand; g Division of Biotechnology, Faculty of Agro-Industry, Chiang Mai University, Chiang Mai 50100, Thailand; h Research Center of Microbial Diversity and Sustainable Utilization, Faculty of Science, Chiang Mai University, Chiang Mai 50200, Thailand

**Keywords:** Extraction, Miang, Tea, Tannase, Antioxidant, Obesity

## Abstract

The aims of this research were to optimize the ultrasonic-assisted enzymatic extraction of polyphenols under Miang and tannase treatment conditions for the improvement of antioxidant activity of Miang extracts via response surface methodology. Miang extracts treated with and without tannase were investigated for their inhibitory effects on digestive enzymes. The optimal conditions for ultrasonic-assisted enzymatic extraction of the highest total polyphenol (TP) (136.91 mg GAE/g dw) and total flavonoid (TF) (5.38 mg QE/g dw) contents were as follows: 1 U/g cellulase, 1 U/g xylanase, 1 U/g pectinase, temperature (74 °C), and time (45 min). The antioxidant activity of this extract was enhanced by the addition of tannase obtained from *Sporidiobolus ruineniae* A45.2 undergoing ultrasonic treatment and under optimal conditions (360 mU/g dw, 51 °C for 25 min). The ultrasonic-assisted enzymatic extraction selectively promoted the extraction of gallated catechins from Miang. Tannase treatment improved the ABTS and DPPH radical scavenging activities of untreated Miang extracts by 1.3 times. The treated Miang extracts possessed higher IC_50_ values for porcine pancreatic α-amylase inhibitory activity than those that were untreated. However, it expressed approximately 3 times lower IC_50_ values for porcine pancreatic lipase (PPL) inhibitory activity indicating a marked improvement in inhibitory activity. The molecular docking results support the contention that epigallocatechin, epicatechin, and catechin obtained via the biotransformation of the Miang extracts played a crucial role in the inhibitory activity of PPL. Overall, the tannase treated Miang extract could serve as a functional food and beneficial ingredient in medicinal products developed for obesity prevention.

## Highlights

• Ultrasonic-assisted enzymatic extraction improved polyphenol and flavonoid contents.
• Ultrasonic-assisted enzymatic extraction promoted the release of gallated catechins.
• Tannase treated Miang extract exhibited high antioxidant activity.
• Tannase treated Miang extract could potentially inhibit digestive enzymes.

## 1 Introduction

Obesity is defined as the abnormal or excessive accumulation of fat that could impair health. This would further support the contention that the fundamental cause of obesity is an energy imbalance between the number of calories consumed and the number of calories expended [1]. It is considered an important risk factor for chronic diseases such as cardiovascular disease, hypertension, hyperlipemia, type 2 diabetes, fatty liver, heart disease, and certain cancers [2], [3]. Accordingly, a trigger for obesity and its associated comorbidities could be intricately linked to an increase in reactive oxygen species (ROS) and a subsequent increase in oxidative stress [4]. Pharmacological and surgical interventions are often employed in strategies administered to prevent obesity; however, they have been associated with a fairly high cost as well as a range of negative effects and potentially hazardous side effects. The development of nutrient digestion and absorption inhibitors to reduce the degree of energy intake through gastrointestinal mechanisms is one of the most promising strategies in the treatment of obesity [5]. Yet, attempts to find a variety of natural products that can inhibit digestive enzymes, along with those that possess antioxidant activity, have received a considerable amount of attention.

Tea (*Camellia sinensis* (L.) Kuntze) is the most widely consumed plant-based beverage in the world. It is commonly known as a rich source of polyphenols. Catechins, the main polyphenols in tea, are considered potentially beneficial biological substances for health and well-being with regard to their antioxidant activity, anti-inflammatory activity, effect on cancer prevention, and regulation of lipid metabolisms [6]. Miang is a traditional fermented tea leaves of *C. sinensis* var. *assamica*, which is typically found in northern Thailand. Miang consists of important biological substances and microorganisms that include tannins, catechins and their derivatives, and organic acids, as well as certain potential probiotic microorganisms [7], [8], [9], [10]. Currently, Miang extracts have exhibited antimicrobial, antioxidant, and anti-inflammatory activities [7], [11]; however, other beneficial health promoting effects attributed to the Miang extract would require further investigation. The non-filamentous fungi growth-based process (NFP) used to produce Miang resulted in considerably higher polyphenol content than the Miang produced via the filamentous fungi growth-based process (FFP) [11]; however, higher polyphenol content and epicatechin content were considerably lower than in three other well-known tea beverages namely green, black, and oolong teas [12], [13]. Based on the outcomes of a previous study, the polyphenol contents of the NFP-Miang ranged from 30 to 35 mg/g dry weight (dw) and the major catechins were epicatechin gallate (ECG), epigallocatechin (EGC), gallocatechin (GC), catechin (C), and epicatechin (EC), while epigallocatechin gallate (EGCG) and gallocatechin gallate (GCG) were present in low amounts at approximately 1 mg/g dw [14].

To further utilize and apply the important bioactive compounds present in low contents at catechins and derivatives, it is inevitable that an appropriated extraction method must be established. The effective extraction of bioactive compounds in tea is dependent upon pH, extraction time and temperature, and solubility. Moreover, the extraction technique can directly influence rate, yield, and purity of the compounds of interest. Four potential extraction techniques have been previously proposed, i.e., solvent-based extraction, microwave-assisted water extraction, ultrasonic extraction, and chemical extraction. Solvent-based and chemical extractions require further steps for solvent removal, whereas exposure to high temperatures during the microwave-assisted extraction process can cause degradation of some bioactive compounds [15]. Notably, ultrasonic-assisted enzymatic extraction can overcome these limitations. For the enhancement of antioxidant activity in tea, some tannases have been found to be able to transform the catechins present in the tea into more active forms [16], [17]. However, only tannase with high substrate specificity towards gallated catechins should be considered. Tannase from *Sporidiobolus ruineniae* A45.2 isolated from Miang is a thermostable enzyme [18] that exhibits high specificity toward gallated catechins (data not shown), yet it was found to be suitable for the biotransformation of Miang extracts in this study. The aims of this research study were to optimize the ultrasonic-assisted enzymatic extraction of the polyphenols present in Miang and to optimize the necessary conditions for improvement of antioxidant activity of the Miang extract. Furthermore, the Miang extracts with and without treatment of tannase were investigated for their inhibitory effects on digestive enzymes. The results of this research could be used to support the applicability of utilizing Miang extracts in the promotion of functional foods and as a potential component in the development of obesity prevention substances.

## 2 Materials and methods

### 2.1 Chemicals and culture media

Food grade cellulase (within a pH range of 3.0 to 6.5 and a temperature range of 35 to 75 °C), xylanase (within a pH range of 4.0 to 9.0 and a temperature range of 25 to 75 °C), and pectinase (within a pH range of 3 to 6.5 and a temperature range of 35 to 75 °C) were purchased from Winovazyme (Beijing, China). Porcine pancreatic α-amylase (with an optimal temperature of 20 °C and an optimal pH of 7.4), porcine pancreatic lipase (with an optimal temperature of 37 °C and an optimal pH of 7), and lysozyme were purchased from Sigma Aldrich (St. Louis, MO, USA). Tannic acid, methyl gallate, gallic acid, rhodanine, 2,2-diphenyl-1-picrylhydrazyl (DPPH), 2,2′-azino-bis (3-ethylbenzothiazoline-6-sulfonic acid) (ABTS), Trolox (6-hydroxy-2,5,7,8-tetramethylchroman-2-carboxylic acid), potassium sulfate, quercetin, and Folin-Ciocalteu’s phenol reagent were all of analytical grade and of the highest quality available from Sigma-Aldrich. High-performance liquid chromatography (HPLC) grade standard (-)-epigallocatechin gallate (EGCG), (-)-epicatechin gallate (ECG), (-)-epigallocatechin (EGC), (-)-epicatechin (EC), (-)-gallocatechin gallate (GCG), (-)-catechin gallate (CG), (-)-gallocatechin (GC), (+)-catechin (C), caffeine, and gallic acid (GA) were all purchased from Sigma. All chemicals used for antioxidant activity assays and enzyme production were of analytical grade and were obtained from RCI Labscan (Bangkok, Thailand). The medium ingredients used in this study, such as agar, yeast extract, and malt extract, were all purchased from HiMedia (Nashik, India).

### 2.2 Microorganism, culture conditions, and production of tannase

A single colony of *Sporidiobolus ruineniae* A45.2 was inoculated in yeast extract-malt extract broth (YMB) (3 g/L yeast extract, 3 g/L malt extract, 10 g/L glucose) and incubated at 30 °C on a 150-rpm rotary shaker for 24 h. Accordingly, 10% (v/v) of inoculum was transferred to YMB supplemented with 1% (w/v) filtered sterile tannic acid and incubated at the same conditions as have been described above. After 48 h of cultivation, the culture was harvested by centrifugation. Cell pellets were washed with 20 mM sodium phosphate buffer pH 6.5 supplemented with 0.1% (v/v) Triton X-100 to remove any gallic acid attached to the yeast cell wall and the residual tannic acid. The cell pellets were then suspended with 20% (w/v) sucrose in 30 mM Tris-HCl pH 8.0. To release the tannase associated with the cell envelope [19], the suspension was supplemented with lysozyme solution prepared in 100 mM EDTA pH 7.3 to yield a final concentration of 0.1 mg/mL of lysozyme prior to being incubated on ice for 40 min. The lysozyme-EDTA treated suspension was centrifuged at 17,350 × g for 15 min at 4 °C. The supernatant was then dialyzed against 20 mM sodium phosphate buffer pH 7.0 at 4 °C until equilibrium was reached. The resulting dialyzed enzyme was then used in further experiments.

Tannase activity was determined according to the method described in a previous study [20] with slight modifications. Briefly, 50 μL of the enzyme solution was mixed with 50 μL of the substrate (12.5 mM methyl gallate in 100 mM sodium phosphate buffer pH 6.5). The reaction was carried out at 37 °C for 20 min. Then, 60 μL of 0.667% (w/v) methanolic rhodanine solution was added into the reaction mixture to stop the reaction and to detect the release of gallic acid from tannic acid. After a 5 min period of incubation at room temperature (25 °C), a pinkish purple color was visualized by adding 40 μL of 0.5 M KOH and the mixture was left at room temperature for 5 min. Finally, 800 μL of distilled water was added, the mixture was vigorously mixed, and absorbance was measured at 520 nm. One unit of tannase was defined as the amount of enzyme that released 1 μmol of gallic acid in 1 min under the assay conditions.

### 2.3 Preparation of Miang

Astringent Miang (7-day fermentation period) made from young tea leaves of *C*. *sinensis* var. *assamica* was purchased from tea plantations located in Mae Taeng District, Chiang Mai Province, Thailand (N19.19441, E98.77654). It was dried using a vacuum dryer at 50–60 °C prior to being ground and sieved through a 30-mesh screen for further use.

### 2.4 Optimization for the ultrasonic-assisted enzymatic extraction of total polyphenol (TP) and total flavonoid (TF) contents

In this study, only water was employed as an extraction solvent due to the fact that previous studies have shown its importance as an environmentally friendly solvent with high efficiency in the recovery of antioxidant phytochemicals [21]. Plackett and Burman design (PBD) was used to screen for the most effective factors that positively influenced the extraction efficiency as follows: cellulase (1–10 U/g dw tea), xylanase (1–10 U/g dw tea), pectinase (1–10 U/g dw tea), temperature (45–65 °C), and time (10–50 min). All enzymes used in this experiment, including cellulase, xylanase, and pectinase, were standardized by determining the enzyme activity according to the method previously described by Kanpiengjai et al. [22] with some modifications. The definition of each enzyme is as follows. One unit of cellulase was defined as the amount of the enzyme that catalyzed the hydrolysis of cellulose (0.5% w/v, pH 5.5) to release 1 μmol of reducing sugars equivalent to glucose in 1 min under assay conditions (pH 5.5, 50 °C, 10 min). One unit of xylanase was defined as the amount of the enzyme that catalyzed the hydrolysis of xylan from birchwood (0.5% w/v, pH 5.5) to release 1 μmol of reducing sugars equivalent to xylose in 1 min under the assay conditions (pH 5.5, 50 °C, 10 min). One unit of pectinase was defined as the amount of the enzyme that catalyzed the hydrolysis of citrus pectin (0.5% w/v, pH 5.5) to release 1 μmol of reducing sugars equivalent to galacturonic acid in 1 min under assay conditions (pH 5.5, 50 °C, 10 min).

**Fig. 1:**
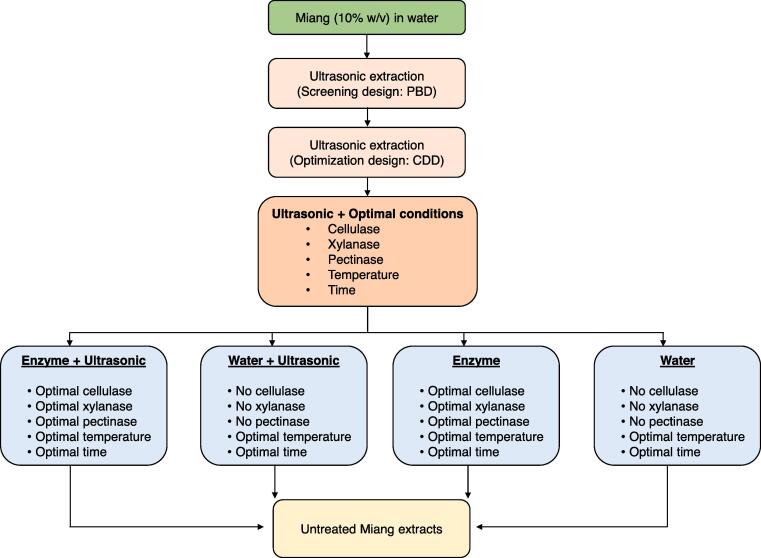
A schematic diagram for extraction of polyphenols and flavonoids from Miang.

Based on the PBD matrix, 12 treatment combinations were generated with three center points for the extraction of Miang. A total of 0.5 g of Miang was mixed with 5 mL of distilled water prior to enzymes being added and a period of incubation being initiated at a specific temperature and time. All treatment combinations were performed in a 40 kHz ultrasonic bath with 150 W ultrasonic power (GT SONIC-D6, GT-Sonic RoHS, Shanghai Tense Electronical Equipment Co., Ltd., China). The ultrasonic bath was connected with a low temperature water circulator (B.E. Marubishi Co. Ltd., Tokyo, Japan) in order to avoid thermal variance due to ultrasonic thermal effect. After the extraction process, the mixture was centrifuged at 17,350 × g. The clear supernatant was immediately collected for determination of TP and TF contents that corresponded to each treatment combination. The experimental responses were fitted with the first-order model. The impact of a range of factors affecting the response variables and model reliability was evaluated by analysis of variance (ANOVA) and regression analysis, respectively. The factors whose *p*-values were less than 0.05 were considered significant factors and were further optimized by central composite design (CCD). In CCD, five-level coded values of each screened factor were established including factorial points (– 1, + 1), axial points (– α, + α), and the center point (0). The number of generated treatment combinations are dependent upon the number of screened factors. Extraction of Miang was also performed in an ultrasonic bath under the same conditions that had previously been described. The TP and TF contents were determined to achieve the response variables that fit with the second-order polynomial model. ANOVA and regression analysis were also performed as has been described above. Finally, the regression equation together with the 3D-contour plots were used to predict the optimal values of the experimental factors for the highest TP and TF contents. In addition, validation of the predicted value was performed to ensure optimal model fitting. Under optimal conditions, the effects of ultrasonic or enzyme treatment on the extraction of TP and TF were compared with the control (no ultrasonic and no enzyme treatments). The profiles of gallic acid, caffeine, and catechins, as well as their contents, were determined by high-performance liquid chromatography (HPLC). Fig. 1 summarizes the Miang extraction procedure for untreated Miang extracts.

### 2.5 Optimization for ultrasonic-assisted enzymatic treatment of Miang extract

The enhancement of antioxidant activity of the Miang extract was investigated by employing the ultrasonic-assisted tannase treatment. After the optimal conditions were established for the extraction of Miang, the obtained extract was immediately treated with tannase derived from *S. ruineniae* A45.2. Here, optimal levels of tannase, temperature, and time were investigated using CCD. Five values for tannase (500, 898, 750, 601, and 1000 mU/g tea), temperature (30, 34, 40, 46, and 50 °C), and time (5, 10, 17.5, 25, and 30 min) (Table 1S) were set in order to generate 14 treatment combinations with six center points, thus resulting in a set of 20 conditions being established for the treatment of Miang extract with tannase. After the treatment of Miang extract with tannase, the DPPH and ABTS radical scavenging activities obtained from each treatment combination were determined and expressed as μmol Trolox equivalent (TE)/g dw. Statistical analyses of the response variables were performed as has been previously described. Miang extract obtained from different extraction methods (Section 2.4) was treated with tannase by employing the optimal conditions. The contents of catechins, caffeine, and gallic acid obtained from all treated Miang extracts were determined by HPLC.

### 2.6 Determination of TP content

The TP content of the extract was determined by employing the Folin-Ciocalteu method according to the procedure described in a previous study [23]. A sample of 0.80 mL was mixed with 0.05 mL of Folin-Ciocalteu reagent. After 1 min, 0.150 mL of 20% (w/v) sodium carbonated was added. The mixture was then allowed to stand at room temperature in the dark for 120 min prior to the absorbance being measured at 750 nm. Gallic acid (GA) was used as the standard. TP content was expressed as mg GA equivalent (GAE)/g dry weight (dw) of Miang.

### 2.7 Determination of TF content

The TF content of the extract was determined by employing the aluminium chloride method according to the procedure explained in a previous study [24]. A total of 200 μL of Miang extract was mixed with 40 μL of 10% (w/v) AlCl_3_ solution prepared in methanol, 40 μL of 1 M potassium acetate, and 1.12 mL of distilled water. The mixture was incubated for 30 min at room temperature prior to the absorbance being measured at 415 nm. Quercetin (Q) was used as the standard. The TF content was expressed as mg quercetin equivalent (QE)/g dw.

### 2.8 Assays of antioxidants

DPPH assay was performed by mixing a sample (0.25 mL) with 2.25 mL of freshly prepared 40 mg/L methanolic DPPH. The reaction was allowed to stand in the dark at room temperature (25 °C). A decrease in absorbance at 517 nm was determined after 30 min of incubation. The concentration of the sample that produced a degree of inhibition between 20% and 80% of the blank absorbance was determined and adapted. Radical scavenging activity was expressed as the concentration of the extract required for inhibition of the initial concentration of DPPH by 50% (IC_50_) under specified experimental conditions. DPPH radical scavenging activity was expressed as μmol TE/g dw.

ABTS radical scavenging activity was then performed. ABTS of 0.0384 g was dissolved in 10 mL of deionized water. Then, 5 mL of the solution was mixed with 88 μL of 140 mM potassium persulfate and adjusted to 25 mL with deionized water in a volumetric flask for further experimentation. An ABTS solution of 1.75 mL was mixed thoroughly with 0.25 mL of the sample. The reaction was allowed to stand in the dark at room temperature. A decrease in absorbance at 734 nm was determined after 30 min of incubation. The concentration of the sample that produced between 20% and 80% inhibition of the blank absorbance was then determined and adapted. Radical scavenging activity was expressed as the concentration of the extract required for inhibition of the initial concentration of ABTS by 50% (IC_50_) under specified experimental conditions. ABTS radical scavenging activity was expressed as μmol TE/g dw.

### 2.9 Determination of gallic acid, caffeine, and catechins by HPLC

The gallic acid, caffeine, and catechin contents were determined using an HPLC system equipped with an Inertsil ODS-3 (5 μm, 25 × 0.46 cm ID) (GL Sciences Inc., Tokyo, Japan) and a UV–Vis detector. The mobile phase consisted of 0.05% phosphoric acid (solvent A) and acetonitrile (solvent B). Initially, the column was equilibrated with a mixture of 90% solvent A and 10% solvent B. Separation was achieved with a linear gradient program as follows: 25% solvent B and 75% solvent A for 15 min, then increased to 60% solvent B and 40% solvent A for 10 min. Flow rate and separation temperature were set up at 1 mL/min and 25 °C, respectively. The catechins were detected by absorbance at 280 nm. Finally, gallic acid, caffeine, and catechin contents were calculated and expressed as mg/g dw.

### 2.10 Effect of Miang extracts on porcine pancreatic α-amylase activity

The porcine pancreatic α-amylase (PPA) activity was determined by measurement of soluble starch retained after the enzyme – soluble starch reaction. Briefly, 100 μL of 1.5% (w/v) soluble starch prepared in 100 mM sodium phosphate buffer pH 6.5 was mixed with 100 μL of the PPA and 300 μL of the same buffer or the Miang extract and then incubated at 37 °C. After 10 min, the reaction was stopped by adding 0.5 mL of 1 M HCl. An aliquot amount of the mixture (200 μL) was mixed with 800 μL of iodine solution (0.3 g/L I_2_, 6 g/L KI). A degree of absorbance at 620 nm was then measured in the reaction. One unit (U) of PPA was defined as the amount of the enzyme required to hydrolyze 1 μg soluble starch in 1 min under the standard assay conditions. To determine the inhibition percentage of Miang against the PPA, PPA activity in the reaction was initially set up as 128 U. The PPA activity was then assayed in the presence of various concentrations of the Miang extract as the inhibitor and compared to that without the presence of an inhibitor. The IC_50_ value was determined from the regression curve and expressed as g/100 mL of the Miang extract.

### 2.11 Effect of Miang extracts on porcine pancreatic lipase activity

Based on the findings of a previous study [25], lipase activity was determined by measuring the free fatty acid released after the enzyme – olive oil reaction. Briefly, 3 mL of olive oil was mixed with 2.5 mL of deionized water or the Miang extract, 1 mL of 100 mM sodium phosphate buffer pH 6.5, and 0.5 mL of Tween 80. The mixture was then vigorously mixed using a magnetic stirrer for 15 min to obtain an emulsion. The porcine pancreatic lipase (PPL) (100 U) was added to the emulsified mixture and incubated on a 150-rpm rotary shaker at 37 °C for 30 min. At the end of the incubation period, 3 mL of 95% ethanol was added prior to the mixture, which was then titrated with 50 mM NaOH using an automatic potentiometric titrator. The end point for the titration was set at pH 9.0. One unit of lipase activity was defined as the amount of enzyme that catalyzed the hydrolysis of triglycerides to release 1 microequivalent of fatty acids in 1 min under standard assay conditions. To determine the percentage of the inhibitory concentration of Miang against PPL, the PPL activity in the reaction was initially set up as 200 U. The PPL activity was assayed in the presence of various concentrations of the Miang extract as the inhibitor and compared to that without the presence of an inhibitor. The IC_50_ value was determined from the regression curve and expressed as g/100 mL of Miang extract.

### 2.12 Molecular docking analysis

For protein structure preparation, the crystal structure of the porcine pancreatic lipase-colipase in a complex with tetraethylene glycol monooctyl ether (TGME) (PDB ID: 1ETH) [26] was retrieved from the RCSB Protein Data Bank. The TGME molecule was separated from the protein structure using Discovery Studio Client v.21.1.0 (Dassault Systèmes Biovia Corp.). The protein was converted from ‘pdb’ to a ‘pdbqt’ format using Python script (Prepare_receptor4.py) the AutoDock Tool (ADT) and the metal charges were then automatically calculated (e.g., zinc ion = +2.0). Resolution of the three-dimensional grid box (*x*, *y*, and *z*) was set at 26 × 34 × 36 for the active pocket [27] and 22 × 30 × 28 for the catechin binding pocket [28] with a grid spacing of 0.375 Å. The center of the grid was set to 56.658, 47.892, and 122.042 Å for the *×*, *y*, and *z* dimensions of the active site and 63.305, 27.761, and 149.683 Å for the *×*, *y*, and *z* dimensions of the catechin binding site, respectively.

For ligand structure preparation, catechin and derivative structures were sketched as a ‘mol2′ file using Discovery Studio Client v.21.1.0. The ligand structures were subsequently assigned according to atom type, while energy optimization was performed using the steepest descent algorithm in the MMFF94 force field via the Avogadro v.1.2.0 program. The ligands were then converted from ‘mol2′ into a ‘pdbqt’ format using MGLTools version 1.5.7.

All molecular docking experiments were performed using AutoDock Vina [29] on a Linux operating platform. The docking parameters were set as follows: exhaustiveness = 20 and 50 for the active site and the catechin binding site, respectively, and an energy range of 2 kcal/mol. The best docking conformation for each complex was selected from the output file based on the position and intermolecular interactions in the active pocket and in the catechin binding site of the porcine lipase-colipase protein. Interactions between the proteins and ligands were visualized by Discovery Studio Clients v.21.1.0.

### 2.13 Statistical design and analysis

In this study, PBD, CCD, ANOVA, and regression analyses were performing using Design Expert software version 7.0 (Stat-Ease Corporation, MN, USA). All experiments were performed in duplicate. The results are presented as values of mean ± standard deviation (SD). Data analysis of the mean values was performed based on a full factorial complete randomized design (CRD). Multiple comparison tests were performed based on all pairwise comparisons using Tukey’s HSD test at a confidence level of 95%. The paired *t*-test was used to compare the results from the experimental and control groups. For comparison tests, all analyses were carried out using the Statistix software version 8.0 (Analytical software, FL, USA). A probability value of *p* < 0.05 was considered significant.

## 3 Results

### 3.1 Extraction of polyphenols and flavonoids from Miang

PBD was used to evaluate the most significant variables influencing the extraction of TP and TF from Miang. For the experimental design matrix of PBD, the experimental and predicted values for the extraction of polyphenols and flavonoids from Miang are shown in Table 1. The maximal TP and TF contents were 128 mg GAE/g dw and 4.81 mg QE/g dw, respectively. The experimental TP and TF contents were well-fitted with the least square linear regression model, wherein the significance of the model fit values (*p* < 0.05) was aligned with the R^2^-values and the adjusted R^2^-values that were higher than 0.90. Temperature and time were the significant factors (*p* < 0.05) that enhanced extractability of TP and TF in contrast to cellulase and xylanase, which were found to have a significantly negative effect on the extraction of the compounds.

**Table 1:** Experimental design matrix of PBD and response variables for screening of the most significant factors enhancing ultrasonic-assisted enzyme extraction of polyphenols and flavonoids from Miang.

Run	A: Cellulase	B: Xylanase	C: Pectinase	D: Temperature (°C)	E: Time (min)	Total polyphenols (mg GAE/g dw)		Total flavonoids (mg QE/g dw)	
						Actual	Predicted	Actual	Predicted
1	10 (+1)	10 (+1)	1 (-1)	65 (+1)	50 (+1)	111.09	115.44	4.36	4.40
2	1 (-1)	10 (+1)	10 (+1)	45 (-1)	50 (+1)	118.64	119.67	3.14	3.19
3	10 (+1)	1 (-1)	10 (+1)	65 (+1)	10 (-1)	118.18	120.06	4.08	4.14
4	1 (-1)	10 (+1)	1 (-1)	65 (+1)	50 (+1)	126.55	123.67	4.59	4.56
5	1 (-1)	1 (-1)	10 (+1)	45 (-1)	50 (+1)	128.00	128.20	3.53	3.57
6	1 (-1)	1 (-1)	1 (-1)	65 (+1)	10 (-1)	127.73	126.97	4.81	4.74
7	10 (+1)	1 (-1)	1 (-1)	45 (-1)	50 (+1)	117.73	118.65	4.05	3.85
8	10 (+1)	10 (+1)	1 (-1)	45 (-1)	10 (-1)	107.91	104.89	3.10	3.26
9	10 (+1)	10 (+1)	10 (+1)	45 (-1)	10 (-1)	106.73	106.21	2.96	2.82
10	1 (-1)	10 (+1)	10 (+1)	65 (+1)	10 (-1)	118.73	119.76	4.01	3.92
11	10 (+1)	1 (-1)	10 (+1)	65 (+1)	50 (+1)	128.91	125.29	4.27	4.34
12	1 (-1)	1 (-1)	1 (-1)	45 (-1)	10	120.27	121.65	3.73	3.81
13	5.5 (0)	5.5 (0)	5.5 (0)	55 (0)	30 (0)	122.36	120.82	4.05	4.05
14	5.5 (0)	5.5 (0)	5.5 (0)	55 (0)	30 (0)	120.82	120.82	4.06	4.05
15	5.5 (0)	5.5 (0)	5.5 (0)	55 (0)	30 (0)	119.27	120.82	4.03	4.05

On the other hand, pectinase had no effect on the extraction of polyphenols, but it did strongly affect the extraction of flavonoids (Table 2). Based on the results, temperature and time were selected for further optimization. Other factors were fixed at their low levels in terms of their effects and their relevant significant differences (1 U/g cellulase, 1 U/g xylanase, and 1 U/g pectinase).

**Table 2:** Regression of coefficients and ANOVA of the first-order model for total polyphenols and total flavonoids in PBD.

I) Total polyphenols						
Source	Coefficient Estimate	Sum of Squares	df	Mean Square	*F*-Value	*p*-Value Prob > *F*
Model	110.0943	593.3974	5	118.6795	14.9602	0.0007*
A-Cellulase	−0.9141	203.0640	1	203.0640	25.5973	0.0010*
B-Xylanase	−0.9478	218.2982	1	218.2982	27.5177	0.0008*
C-Pectinase	0.1465	5.2128	1	5.2128	0.6571	0.4410
D-Temperature	0.2659	84.8492	1	84.8492	10.6957	0.0113*
E-Time	0.1307	81.9731	1	81.9731	10.3332	0.0123*
Curvature		6.2492	1	6.2492	0.7877	0.4007
Residual		63.4642	8	7.9330		
Lack of Fit		58.6873	6	9.7812	4.0953	0.2092
Pure Error		4.7769	2	2.3884		
Cor Total		663.1107	14			

Std. Dev.	2.8166		R^2^		0.9034	
Mean	119.5273		Adjusted R^2^		0.8430	
C.V. %	2.3564		Predicted R^2^		0.6298	
PRESS	245.4972		Adequate Precision		12.1113	

II) Total flavonoids						
Source	Coefficient Estimate	Sum of Squares	df	Mean Square	*F*-Value	*p*-Value
						Prob > *F*

Model	1.7618	3.846	5	0.7692	50.9451	< 0.0001*
A-Cellulase	−0.0183	0.081	1	0.0810	5.3672	0.0492*
B-Xylanase	−0.0425	0.440	1	0.4398	29.1272	0.0006*
C-Pectinase	−0.0487	0.577	1	0.5767	38.1934	0.0003*
D-Temperature	0.0467	2.621	1	2.6211	173.6044	< 0.0001*
E-Time	0.0052	0.127	1	0.1273	8.4335	0.0198*
Curvature		0.064	1	0.0636	4.2095	0.0743
Residual		0.121	8	0.0151		
Lack of Fit		0.120	6	0.0200	60.3404	0.0164*
Pure Error		0.001	2	0.0003		
Cor Total		4.030	14			

Std. Dev.	0.1229		R^2^		0.9695	
Mean	3.9170		Adjusted R^2^		0.9505	
C.V. %	3.1369		Predicted R^2^		0.8804	
PRESS	0.4820		Adequate Precision		22.8780	

* Significant difference at *p* < 0.05.

For CCD optimization, temperature (A) and time (B) were extended to have broader ranges than those in PBD. The experiments were performed to achieve the experimental TP and TF values (Table 3). The ANOVA results confirmed the PBD in terms of the significant effect of temperature and time on the extraction of TP and TF from Miang (Table 2S). The models for extraction of TP and TF were significantly fitted with the second-order model, thus establishing regression equations based on coded values to predict the extractability of TP and TF from Miang as follows:

$$\text{Total}\;\text{polyphenols}\;\;\left(\text{mg}\;\text{GAE}/\text{g}\;\text{dw}\right)\;=\;127.82\;+\;6.31\text{A}\;+\;1.79\text{B}-\;0.32\text{AB}\;-\;3.02{\text{A}}^{2}-\;4.75{\text{B}}^{2}$$

$$\text{Total}\;\text{flavonoids}\;\left(\text{mg}\;\;\text{QE}/\text{g}\;\text{dw}\right)\;=\;4.51\;+\;0.74\text{A}\;+\;0.14\text{B}\;+\;0.0715\text{AB}\;-\;0.30{\text{A}}^{2}\;-\;0.25{\text{B}}^{2}$$

**Table 3:** Experimental design matrix of CCD and experimental and predicted values of TP and TF for quantitative determination of optimal temperature and time for ultrasonic-assisted enzymatic extraction from Miang.

Run	A: Temperature (°C)	B: Time (min)	Total polyphenols (mg GAE/g dw)		Total flavonoids (mg QE/g dw)	
			Experimental	Predicted	Experimental	Predicted
1	45 (-1)	20 (-1)	110.91	111.62	3.03	3.15
2	75 (+1)	20 (-1)	126.45	124.88	4.64	4.50
3	45 (-1)	60 (+1)	116.09	115.84	3.01	3.29
4	75 (+1)	60 (+1)	130.36	127.84	4.91	4.91
5	39 (-1.414)	40 (0)	113.55	112.84	3.12	2.86
6	81 (+1.414)	40 (0)	128.18	130.70	4.83	4.96
7	60 (0)	12 (-1.414)	115.55	115.78	3.78	3.82
8	60 (0)	68 (+1.414)	119.27	120.86	4.39	4.21
9	60 (0)	40 (0)	128.45	127.82	4.48	4.51
10	60 (0)	40 (0)	126.18	127.82	4.48	4.51
11	60 (0)	40 (0)	130.55	127.82	4.52	4.51
12	60 (0)	40 (0)	127.27	127.82	4.55	4.51
13	60 (0)	40 (0)	126.64	127.82	4.54	4.51

**Fig. 2:**
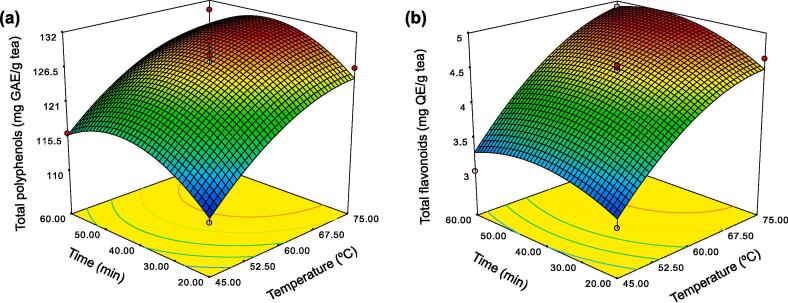
Three-dimensional curves and contour plots demonstrating the effects of time and temperature on extractions of polyphenols (a) and flavonoids (b) derived from Miang.

**Fig. 3:**
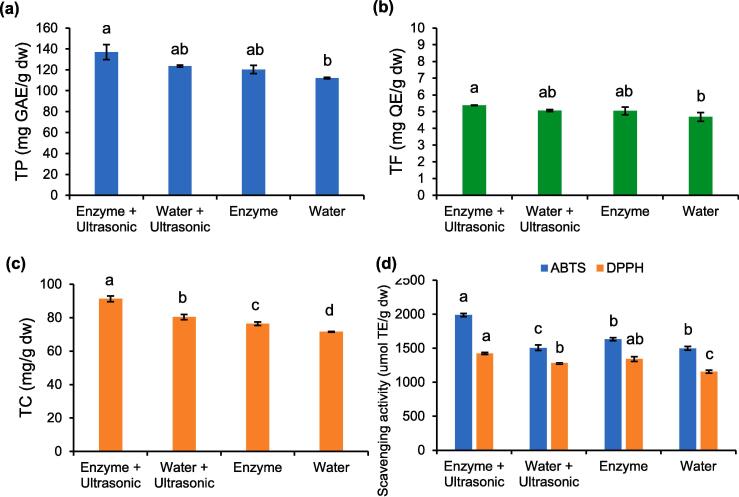
Effects of different extraction methods on extractability of TP (a) and TF (b) derived from Miang.

According to the regression models, response surface plots were employed and are shown in Fig. 2. These models produced acceptable results when *p* < 0.05 with R^2^-values between 0.92 and 0.97. A TP content of 131.12 mg GAE/g dw and a TF content of 4.97 mg QE/g dw were predicted from the regression models and successfully validated at 95.5% (136.91 mg GAE/g dw) and 91.9% (5.38 mg QE/g dw), respectively. This was achieved when Miang was extracted at 74 °C for 45 min by employing the ultrasonic, cellulase, xylanase, and pectinase treatments. Under optimal conditions, the ultrasonic-assisted enzymatic extraction method exhibited the potential to significantly increase TP, TF, and TC contents as well as antioxidant activity (Fig. 3) when compared with other extraction methods including enzyme extraction, ultrasonic extraction, and water extraction.

**Fig. 4:**
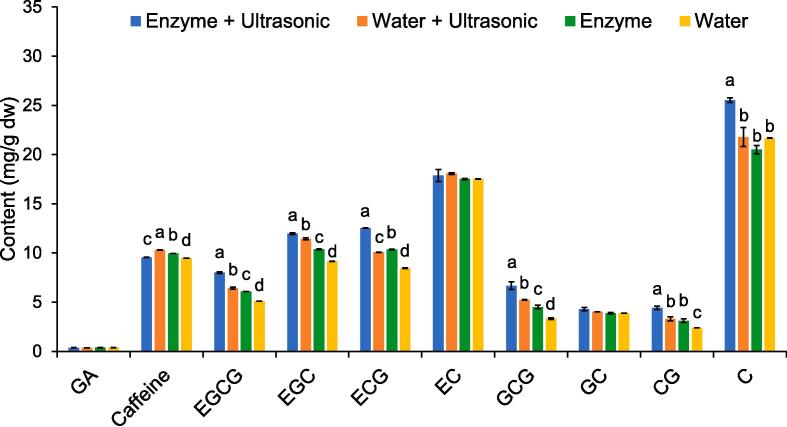
Profile of gallic acid, caffeine, and catechins derived from Miang extract obtained using different extraction methods.

The highest TC content was 91.28 ± 1.76 mg/g dw which included both epicatechins and non-epimer catechins in the following order: C (25.53 ± 0.25 mg/g dw) > EC (17.86 ± 0.60 mg/g dw) > EGC (11.96 ± 0.08 mg/g dw) = ECG (12.53 ± 0.53 mg/g dw) > EGCG (8.00 ± 0.10 mg/g dw) (Fig. 4). In addition, the ultrasonic-assisted enzymatic extraction encouraged the release of significant amounts of gallated catechins.

Different lowercase letters in the columns with the same dept indicate differences in antioxidant activity at *p* < 0.05.

### 3.2 Enhancement of antioxidant activity of Miang extract by yeast tannase

The effect of tannase (A), temperature (B), and time (C) on the antioxidant activity of the Miang extract was evaluated based on the design matrix of CCD (Table 4). Consequently, the experimental ABTS and DPPH radical scavenging activities were achieved.

**Table 4:** Experimental design matrix of CCD and response variables for enhancement of antioxidant activity of Miang extract by administering ultrasonic-assisted tannase treatment.

Run	A: Tannase (mU/g dw)	B: Temperature (°C)	C: Time (min)	Antioxidant activity (μmol TE/g dw)			
				ABTS·		DPPH·	
				Experimental	Predicted	Experimental	Predicted
1	141 (-1)	38 (-1)	10 (-1)	2,189.85	2,162.15	1,498.05	1,532.86
2	409 (+1)	38 (-1)	10 (-1)	2,346.68	2,328.33	1,537.36	1,547.97
3	141 (-1)	62 (+1)	10 (-1)	2,227.71	2,138.52	1,573.61	1,542.59
4	409 (+1)	62 (+1)	10 (-1)	2,406.79	2,356.87	1,619.07	1,590.21
5	141 (-1)	38 (-1)	25 (+1)	2,348.21	2,355.06	1,619.92	1,647.53
6	409 (+1)	38 (-1)	25 (+1)	2,395.60	2,441.73	1,699.76	1,729.52
7	141 (-1)	62 (+1)	25 (+1)	2,361.31	2,336.59	1,659.73	1,647.87
8	409 (+1)	62 (+1)	25 (+1)	2,490.82	2,475.44	1,798.43	1,762.36
9	50 (-1.68)	50 (0)	17.5 (0)	2,160.77	2,220.13	1,625.22	1,613.00
10	500 (+1.68)	50 (0)	17.5 (0)	2,475.08	2,476.63	1,707.99	1,721.99
11	275 (0)	30 (-1.68)	17.5 (0)	2,271.37	2,246.48	1,606.00	1,544.27
12	275 (0)	70 (+1.68)	17.5 (0)	2,169.16	2,254.96	1,516.57	1,580.07
13	275 (0)	50 (0)	5 (-1.68)	2,179.58	2,268.92	1,537.52	1,545.51
14	275 (0)	50 (0)	30 (+1.68)	2,559.26	2,530.83	1,792.92	1,786.70
15	275 (0)	50 (0)	17.5 (0)	2,447.01	2,475.70	1,740.81	1,744.22
16	275 (0)	50 (0)	17.5 (0)	2,440.00	2,475.70	1,764.74	1,744.22
17	275 (0)	50 (0)	17.5 (0)	2,488.96	2,475.70	1,763.70	1,744.22
18	275 (0)	50 (0)	17.5 (0)	2,481.92	2,475.70	1,707.13	1,744.22
19	275 (0)	50 (0)	17.5 (0)	2,496.08	2,475.70	1,720.89	1,744.22
20	275 (0)	50 (0)	17.5 (0)	2,510.67	2,475.70	1,768.34	1,744.22

These values significantly fitted with the second-order model at *p* < 0.05 by establishing two reliable regression models with R^2^-values between 0.87 and 0.90 for the models ABTS and DPPH (Table 3S). The highest antioxidant activity was predicted by applying the following coding equations:

$$\text{ABTS}\cdot \text{scavenging}\;\text{activity}\;\left(\mu \text{mol}\;\text{TE}/\text{g}\;\text{dw}\right)\;=\;2475.70\;+\;76.25\text{A}\;+\;2.52\text{B}+\;77.87\text{C}\;+\;13.04\text{AB}-\;19.88\text{AC}\;+\;1.29\text{BC}-\;45.01{\text{A}}^{2}-\;79.54{\text{B}}^{2}-\;26.81{\text{C}}^{2}$$

$$\text{DPPH}\cdot \text{scavenging}\;\text{activity}\;\left(\mu \text{mol}\;\text{TE}/\text{g}\;\text{dw}\right)\;=\;1744.22\;+\;32.40\text{A}\;+\;10.64\text{B}+\;71.71\text{C}\;+\;8.13\text{AB}+\;16.72\text{AC}\;-\;2.35\text{BC}-\;27.12{\text{A}}^{2}-\;64.36{\text{B}}^{2}-\;27.62{\text{C}}^{2}$$

**Fig. 5:**
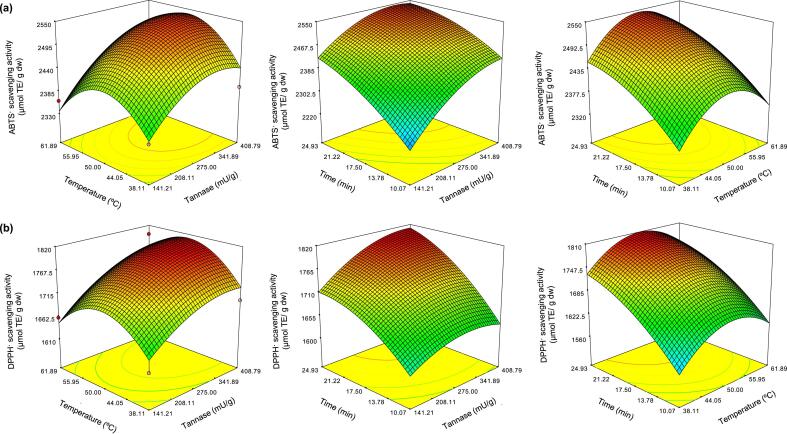
Three-dimensional curves and contour plots demonstrating the effects of tannase, temperature, and time on ABTS (a) and DPPH radical scavenging activities of the Miang extract obtained by the ultrasonic-assisted enzymatic extraction method (b).

The convex shape of the 3D surface plots indicates the optimal conditions for elevation of antioxidant activities (Fig. 5). The regression models predicted the maximal ABTS and DPPH radical scavenging activities of 2,544.87 and 1,809.26 μmol TE/g dw, respectively, when the Miang extract was treated with 360 mU/g dw and the conditions involved 51 °C for 25 min. The predicted values were successfully validated with 2,557.83 ± 59.46 μmol TE/g dw and 1,822.65 ± 23.65 μmol TE/g dw, which resulted in 99% validation, while the antioxidant activity was increased by up to 1.3 times for both the ABTS and DPPH radical scavenging activities.

**Fig. 6:**
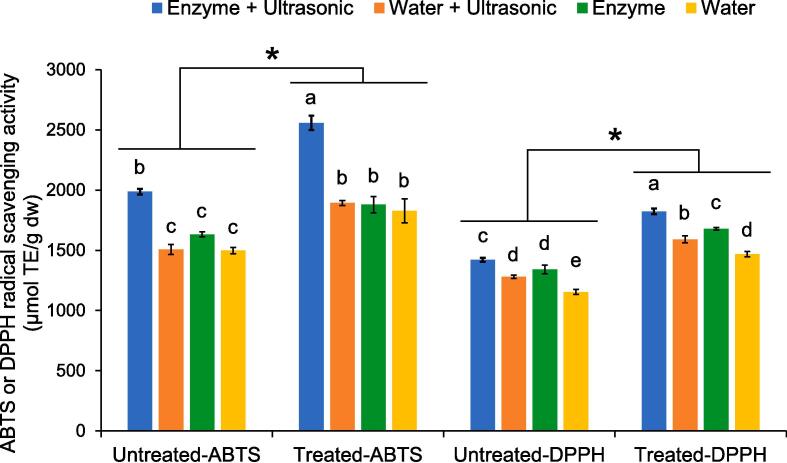
ABTS and DPPH radical scavenging activities of untreated and treated Miang extracts obtained from different extraction methods. * Significant differences in antioxidant activity between untreated and treated Miang extracts. Different lowercase letters in the columns with the same dept indicate differences in antioxidant activity at *p* < 0.05.

When considering the extracts obtained from the different extraction methods, it was confirmed that tannase promoted the antioxidant activity of the Miang extract (Fig. 6).

**Fig. 7:**
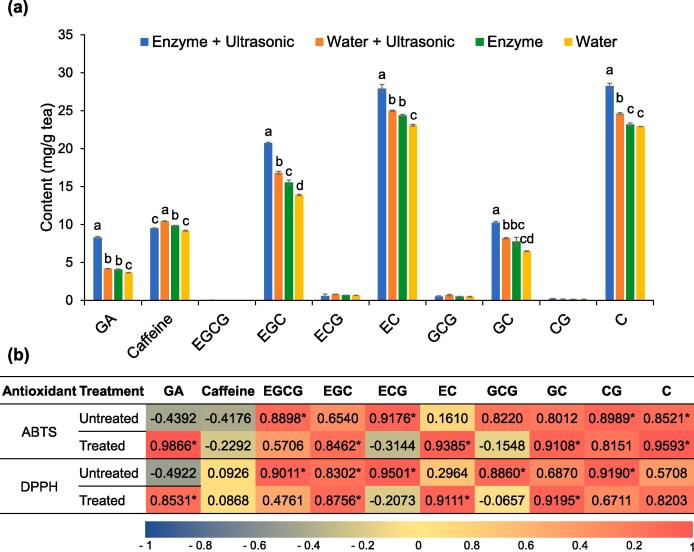
Profile of gallic acid, caffeine, and catechins derived from the tannase treated Miang extracts obtained from different extraction methods (a) and their correlation with ABTS and DPPH radical scavenging activities (b). Different lowercase letters in the columns with the same dept indicate differences in antioxidant activity at *p* < 0.05. * Indicates significant differences at *p* < 0.001.

Additionally, the ultrasonic-assisted enzymatic extraction method yielded the extract with the highest degree of antioxidant activity, which was then improved by employing the tannase treatment. The treated extracts revealed different profiles of catechins (Fig. 7A) when compared with those of the untreated Miang extracts (Fig. 4). The gallated catechins were significantly transformed into non-gallated catechins via the reaction of tannase and consequently released significant amounts of gallic acid. In addition, this phenomenon led to a high titer of non-gallated catechins namely C (28.23 ± 0.36 mg/g dw), EC (27.93 ± 0.53 mg/g dw), and EGC (20.72 ± 0.12 mg/g dw). The example HPLC chromatograms of untreated and treated Miang extracts are presented in Fig. 1S. For the untreated Miang extracts, the ABTS and DPPH radical scavenging activities displayed positive significant correlations (*p* < 0.001) with EGCG (*r* = 0.8898 and *r* = 0.9011), ECG (*r* = 0.9176 and *r* = 0.9501), and CG (*r* = 0.8989 and *r* = 0.9190). After treatment with tannase, positive significant correlations were found between ABTS radical scavenging activity and gallic acid (*r* = 0.9866), C (*r* = 0.9593), EC (*r* = 0.9385), and EGC (*r* = 0.8462) (Fig. 7B). Similarly, positive significant correlations were also found between the DPPH radical scavenging activity and GC (*r* = 0.9195), EC (*r* = 0.9111), EGC (*r* = 0.8756), and gallic acid (*r* = 0.8531), respectively.

### 3.3 *In vitro* inhibitory effect of Miang extract on digestive enzymes

Both untreated and treated Miang extracts obtained from different extraction methods were evaluated for their potential *in vitro* inhibitory effect on PPA and PPL activities. The results indicated that the inhibitory activity of the extract against digestive enzymes was directly influenced by the selected extraction method (Table 5).

**Table 5:** Pancreatic α-amylase and lipase inhibitory activities (IC_50_) of Miang tea extracts obtained from different extraction methods.

Extraction method	α-Amylase inhibitory activity (g/100 mL)		Lipase inhibitory activity (g/100 mL)	
	Untreated	Treated	Untreated	Treated
Enzyme + Ultrasonic	2.17 ± 0.01^a^	6.44 ± 0.03^d^	1.66 ± 0.02^e^	0.46 ± 0.01^a^
Water + Ultrasonic	2.41 ± 0.01^b^	7.12 ± 0.05^e^	2.44 ± 0.01^f^	0.64 ± 0.01^b^
Enzyme	2.45 ± 0.02^bc^	7.49 ± 0.03^f^	2.59 ± 0.04 ^g^	0.71 ± 0.01^c^
Water	2.50 ± 0.03^c^	9.30 ± 0.04 ^g^	2.60 ± 0.03 ^g^	1.03 ± 0.02^d^

Different lowercase letters indicate significant differences in α-amylase inhibitory activity and lipase inhibitory activity at *p* < 0.05.

**Fig. 8:**
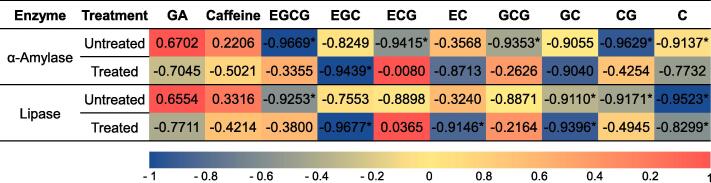
Reverse correlation of gallic acid, caffeine, and catechins obtained from untreated and treated Miang extracts and the inhibitory activities of porcine pancreatic α-amylase and porcine pancreatic lipase. * Indicates significant differences at *p* < 0.001.

The untreated Miang extract exhibited a significantly stronger inhibitory effect on PPA activity than that of the treated extract. Accordingly, its IC_50_ value ranged from 2.17 ± 0.01 to 2.50 ± 0.03 g/100 mL. The most effective fraction for PPA inhibition was expressed by the Miang extract obtained from the ultrasonic-assisted enzymatic extraction, while that of the control displayed the lowest degree of inhibitory activity. On the other hand, the treated Miang extract exhibited a more efficient inhibitory effect on the PPL activity than that of the untreated extract, while its IC_50_ values ranged from 0.46 ± 0.01 to 1.03 ± 0.02 g/100 mL. Reverse correlation analysis indicated that the gallated catechins that were found in the untreated Miang extract were likely to be associated with the best PPA inhibitors (*p* < 0.0001), whereas EGC, EC, and C the most abundant compounds from the treated Miang extract, exhibited the strongest degree of inhibitory activity against PPL (*p* < 0.0001) (Fig. 8).

### 3.4 Molecular docking

Molecular docking analysis was conducted to investigate the mechanisms of interaction between PPL as the receptor and the ligand compound by determining the binding affinity and the binding site of the PPL and the ligand compound. The ligands used in this study were EGCG as the positive control for the PPL inhibitor, as has been suggested in the previous study [30], while the top three major compounds that were found in the untreated and treated Miang extracts included C, EC, EGC, and ECG. The active pocket of PPL is composed of Gly77, Phe78, Ile79, Asp80, Trp86, Tyr115, Ser153, Leu154, Asp177, Pro181, His264, and Leu265 with its catalytic triad includes Ser153, Asp177, and His264 [27]. The epicatechins exhibited the lowest degrees of binding energy in the following order ECG = EC < EGCG < EGC = EC. Alternatively, gallic acid exhibited the highest degree of binding energy at −5.9 kcal/mol (Table 6). The binding energy of these catechins ranged from −9.7 to 9.3 kcal/mol. All tested catechins could effectively interact with the active residues of the PPL via a hydrogen bond and hydrophobic interactions with amino acids at the active pocket of the PPL, along with electrostatic interactions with one of the catalytic triad amino acids.

**Table 6:** Different PPL (1ETH)-ligand complexes and their binding energy and interactions.

1ETH-ligand complexes	Binding energy (kcal/mol)	Hydrogen bonding	Hydrophobic interactions	Electrostatic interactions
1ETH-C	−9.7	GLY77	Phe78, Tyr115, Phe216	His264
1ETH-EC	−9.3	His152	Phe78, Tyr115, Phe216	His264
1ETH-EGC	−9.3	Gly77	Phe78, Tyr115, Phe216	His264
1ETH-ECG	−9.7	Gly77	Phe78, Tyr115, Phe216, Leu265	His264
1ETH-EGCG	−9.4	Gly77, Asp80	Phe78, Ile79, Tyr115, Phe216	His264
1ETH-GA	−5.9	Asp80, Arg257	Ala261, Leu265	–

## 4 Discussion

Despite the fact that Miang has exhibited long and deep-rooted social and cultural integration and relevance with the people of northern Thailand, it is currently less popular among younger generations [31]. Finding a potentially beneficial application for Miang would accordingly conserve the tradition of Miang fermentation. Polyphenols, specifically gallated and non-gallated catechins, are the major bioactive compounds of tea. However, their contents in Miang are relatively lower than those of the most popular varieties of tea including green tea, oolong, and pu-erh [11], [13]. Differences in the polyphenol and catechin contents depend upon the quality of the tea leaves [32] and the tea plantation area from which they were grown [33]. Accordingly, an appropriate extraction technique is a key factor affecting the polyphenol production of the tea. In this study, we attempted to present an interesting extraction strategy, namely ultrasonic-assisted enzymatic extraction, for the extraction of certain bioactive compounds, namely polyphenols and flavonoids. Several studies have revealed that polyphenols, such as tannins, catechins, cyanidin-3-glucoside, and quercetin, can interact with the polysaccharides associated with the plant cell wall, i.e., cellulose, hemicellulose, and pectin via hydrogen bonding, hydrophobic interaction, adsorption, and pi-pi interaction. The network of cellulose and hemicellulose is naturally embedded in the pectin matrix, which is the most complex structure of polysaccharides in the plant cell wall [34], [35]. In order to counteract this process, the effect of carbohydrate-active enzymes on mediating polyphenol-cell wall interactions in Miang would be an alternative strategy to specifically weaken or break down the cell wall structure and significantly contribute to the release of more polyphenol content from Miang, as has been suggested in the previous study [35]. In addition, enzymatic extraction provides several advantages as opposed to conventional extraction methods as follows; mild reaction conditions, processes requiring fewer steps, a substrate specificity that in turn leads to high productivity of bioactive compounds with a high degree of bioavailability and quality, and lower production costs by replacing multiple installations that are needed for the classical extraction processes [36]. The PB results showed that temperature and time were positively significant factors for the extraction of TP and TF contents. Elevated temperatures can increase the solubility but reduce the viscosity and surface tension of the solvent, thus promoting solvent penetration into the matrix and improving the extraction process [37]. Temperature is generally a time dependent factor. Although the addition of the enzyme mixture had a significantly negative effect on the extraction of TP and TF, further results revealed that it is essential to add an enzyme mixture to enhance the degree of extraction efficiency (Fig. 3). It can be determined that a range of each enzyme may be too high to be applied in the extraction. Thus, they were fixed at a level of 1 U/g dw. Optimization for the ultrasonic-assisted enzymatic extraction of Miang was successful with higher titers of TP, TF, and TC contents when compared with the conventional extraction method (no enzyme and ultrasonic treatments). NFP Miang contains TP content of 100 mg GAE/g dw and TC content of 5 mg QE/g dw [14], yet the results of this study indicated 1.5- and 2.5-times higher TP and TC contents, respectively. Green tea made from young tea leaves possessed TP ranging from 108.8 to 323.6 mg/g dw [13], [38], which was higher than that of oolong tea (103.5–297.3 mg/g dw), black tea (130.1–181.7 mg/g dw), and dark tea (78.2–162.9 mg/g dw) [13]. The main classes of flavonoids found in tea are flavanols and flavonols. Although catechins are a flavanol class of flavonoids, it has been observed that the TF content is much lower than the TC content. It can be concluded that the determination of TF by Folin-Ciocalteu does have a limitation when detecting catechins, thus TF may only be referred to as myricetin, quercetin, and kaempferol [39]. Previous studies have reported on the amounts of TF (in terms of total amounts of myricetin, quercetin, and kaempferol) in green tea and black tea in ranges of 4.18–8.95 mg/g dw and 3.0–5.86 mg/g dw, respectively [40], [41], while lower TF contents were found at levels of 2.69 mg/g dw for oolong tea and 1.15 mg/g dw for pu-erh [41]. These reported TF contents are in accordance with the results of this study. The ultrasonic-assisted enzymatic extraction of Miang improved the extractability of TC, particularly gallated catechins both in terms of epicatechins (EGCG and ECG) and non-epimer catechins (GCG and CG). This would affirm that bigger molecules with more hydroxyl groups, like gallated catechins, are retained in greater amounts in the plant cell wall due to hydrogen bonding and hydrophobic interactions [42]. Various catechins in tea possess different antioxidant activities depending upon their type. Based on a molar basis, EGC exhibited the greatest activity, followed by EGCG and GA, and then EC and ECG [23]. Furthermore, the antioxidant activity of EC was comparatively equivalent to that of C [43]. Accordingly, higher antioxidant activity is expected in the resultant concentrations of catechins in the treated Miang extract. Yet, the higher antioxidant activity of the treated Miang extract by tannase could have been due to the presence of high amounts of EGC, EC, C, and GA. In terms of the overall extractability results, it could be stated that carbohydrate-active enzymes can reduce the structural integrity or increase the permeability of the cell wall, thus encouraging acoustic cavitation provided by ultrasonic treatment to disrupt interactions between bioactive compounds and the cell wall [23], [44]. Therefore, the ultrasonic-assisted enzymatic extraction method would be the best extraction method for Miang.

Gallated epicatechins, specifically the EGCG present in tea, display high inhibitory activity against human digestive enzymes, such as amylase, lipase, and trypsin [45], [46], [47], [48], which have been associated with hyperlipidemia and obesity. In this study, the untreated Miang extracts that possessed high amounts of gallated catechins, specifically those that were derived from the optimal conditions for ultrasonic-assisted enzymatic extraction, gave lower IC_50_ values against the PPA than those of the treated extracts. Surprisingly, the treated Miang extract contents consisted of C, EC, and EGC as the major compounds in that respective order, which indicated significantly lower degrees of IC_50_ values against the PPL than the untreated Miang extracts. The molecular docking results that were expressed in terms of binding energy were used to explain the results. Lower binding energy would indicate the formation of a more stable ligand-receptor complex [30]. According to the results obtained from reverse correlation, the EGC, EC, and C were indicated as three active compounds that displayed binding energy that was as low as EGCG, thus indicating their high binding affinity against PPL. From the results, it can be noted that EGCG, EGC, ECG, EC, and C possess a similar degree of binding affinity against PPL. The structural-activity relationship of lipase inhibition achieved by different extract methods was found to contain high phenolic compounds, which has been reported to be dose dependent and dependent upon the type of substrate used [49]. Those outcomes are in agreement with the findings of this study, which indicate that the inhibition of the PPL does require significant amounts of EGC, EC, and C. This could explain why the tannase treated Miang extracts exhibited greater potential than the untreated extracts. In this study, when considering olive oil as the substrate for PPL, it could be stated that EGC, EC, and C could act as competitive inhibitors for the substrate. The effect of the individual catechins on PPL determined by using olive oil as a substrate may be required to further confirm these results. Moreover, the most recent study has revealed that catechins as non-competitive inhibitors could enhance the inhibitory effect of cyanidin-3-glucoside (C3G), a competitive inhibitor on PPL via the catechin-C3G mixture [28]. Further investigations of the synergistic PPL inhibitory activity of catechin-EGC and catechin-EC mixtures have garnered significant interest.

## 5 Conclusion

This study has demonstrated a new strategy for ultrasonic-assisted enzymatic treatment for the extraction of bioactive compounds from Miang and the treatment of tannase to increase the antioxidant potential of the Miang extract. After the statistical optimization step, this ultrasonic-assisted enzymatic extraction method exhibited significantly higher antioxidant activity and resulted in greater amounts of catechins, particularly gallated catechins, than when a conventional method was used. It would therefore be useful for the extraction of phenolic compounds from Miang and is recommended to be applied for phenolic compound extraction from other types of tea. The higher antioxidant activity of the Miang extract could be established by the specific yeast tannase treatment, which then contributes to the formation of non-gallate catechins and gallic acid. Moreover, the treated Miang extract exhibited potential inhibitory effects against the PPA and the PPL. Regarding the inhibitory activity against PPL, the molecular docking results indicate that the non-gallated catechins, namely EGC, EC, and C, may be associated with a reduction in PPL activity. Importantly, further biological properties of the treated Miang extract, such as anti-wrinkle and anti-hypertensive activities, are of significant interest for further investigations.

## CRediT authorship contribution statement

**Nalapat Leangnim:** Methodology, Investigation, Formal analysis, Validation, Writing – review & editing. **Kridsada Unban:** Supervision, Resources. **Patcharapong Thangsunan:** Investigation, Writing – original draft. **Suriya Tateing:** Investigation, Writing – original draft. **Chartchai Khanongnuch:** Supervision, Resources. **Apinun Kanpiengjai:** Conceptualization, Funding acquisition, Methodology, Investigation, Resources, Supervision, Data curation, Writing – original draft, Writing – review & editing.

## Declaration of Competing Interest

The authors declare the following financial interests/personal relationships which may be considered as potential competing interests: Apinun Kanpiengjai reports financial support was provided by Chiang Mai University.

## Data Availability

Data will be made available on request.
