# Supplementary material for: Ultrasonic-assisted enzymatic improvement of polyphenol content, antioxidant potential, and in vitro inhibitory effect on digestive enzymes of Miang extracts
Source: Ultrason Sonochem. 2023 Mar 1;94:106351. doi: 10.1016/j.ultsonch.2023.106351 (PMC9988395; doi:10.1016/j.ultsonch.2023.106351)
Supplement: Supplementary data 1 [file mmc1.docx]

**Supplementary Material**

**Ultrasonic-assisted enzymatic improvement of polyphenol content, antioxidant potential, and *in vitro* inhibitory effect on digestive enzymes of Miang extracts**

Nalapat Leangnim^1,2^, Kridsada Unban^3,4^, Patcharapong Thangsunan^5^, Suriya Tateing^6^, Chartchai Khanongnuch^4,7^, Apinun Kanpiengjai^2,4,8*^

^1^ Program in Biotechnology, The Graduate School, Chiang Mai University, Chiang Mai 50200, Thailand

^2^ Division of Biochemistry and Biochemical Innovation, Faculty of Science, Chiang Mai University, Chiang Mai 50200, Thailand

^3^ Division of Food Science and Technology, Faculty of Agro-Industry, Chiang Mai University, Chiang Mai 50100, Thailand

^4^ Research Center for Multidisciplinary Approaches to Miang, Chiang Mai University, Chiang Mai 50200, Thailand

^5^ Center of Excellence in Fish Infectious Diseases (CE FID), Department of Veterinary Microbiology, Faculty of Veterinary Science, Chulalongkorn University, Bangkok, 10330, Thailand.

^6^ Department of Plant and Soil Sciences, Faculty of Agriculture, Chiang Mai University, Chiang Mai 50200, Thailand

^7^ Division of Biotechnology, Faculty of Agro-Industry, Chiang Mai University, Chiang Mai 50100 Thailand

^8^ Research Center of Microbial Diversity and Sustainable Utilization, Faculty of Science, Chiang Mai University, Chiang Mai 50200, Thailand

___________________________________________________________________________

Corresponding author: Apinun Kanpiengjai

Corresponding author e-mail: ak.apinun@gmail.com, apinun.k@cmu.ac.th

**Table 1S** Coded and actual values of independent factors for generating CCD design matrix to determine optimal conditions for enhancing antioxidant activity of Miang extract.

| Factor | ‒ α (‒ 1.68) | ‒ 1 | 0 | + 1 | + α (+ 1.68) |
| --- | --- | --- | --- | --- | --- |
| Tannase (mU/g dw) | 500 | 898 | 750 | 601 | 1,000 |
| Temperature (ºC) | 30 | 34 | 40 | 46 | 50 |
| Time (min) | 5 | 10 | 17.5 | 25 | 30 |

**Table 2S** Regression of coefficients and ANOVA of the second-order model for quantitative determination of optimal temperature and time for ultrasonic-assisted enzymatic extraction of TP and TF from Miang.

| I) Total polyphenols |  |  |  |  |  |  |
| --- | --- | --- | --- | --- | --- | --- |
| Source | Coefficient | Sum of | df | Mean | *F*-Value | *p*-Value |
|  | Estimate | Squares |  | Square |  | Prob > *F* |
| Model | 127.8182 | 543.0132 | 5 | 108.6026 | 24.4874 | 0.0003* |
| A-Temperature | 6.3146 | 318.9975 | 1 | 318.9975 | 71.9265 | < 0.0001* |
| B-Time | 1.7953 | 25.7836 | 1 | 25.7836 | 5.8136 | 0.0467* |
| AB | -0.3182 | 0.4050 | 1 | 0.4050 | 0.0913 | 0.7713 |
| A^2^ | -3.0227 | 63.5609 | 1 | 63.5609 | 14.3315 | 0.0068* |
| B^2^ | -4.7500 | 156.9565 | 1 | 156.9565 | 35.3901 | 0.0006* |
| Residual |  | 31.0453 | 7 | 4.4350 |  |  |
| Lack of Fit |  | 18.8304 | 3 | 6.2768 | 2.0555 | 0.2488 |
| Pure Error |  | 12.2149 | 4 | 3.0537 |  |  |
| Cor Total |  | 574.0585 | 12 |  |  |  |
| Std. Dev. | 2.1060 |  |  | R^2^ | | 0.9459 |
| Mean | 123.0350 |  |  | Adjusted R^2^ | | 0.9073 |
| C.V. % | 1.7117 |  |  | Predicted R^2^ | | 0.7335 |
| PRESS | 152.9911 |  |  | Adequate Precision | | 13.3399 |
| II) Total flavonoids |  |  |  |  |  |  |
| Source | Coefficient | Sum of | df | Mean | *F*-Value | *p*-Value |
|  | Estimate | Squares |  | Square |  | Prob > *F* |
| Model | 4.5122 | 5.5156 | 5 | 1.1031 | 32.2332 | 0.0001* |
| A-Temperature | 0.7419 | 4.4036 | 1 | 4.4036 | 128.6731 | < 0.0001* |
| B-Time | 0.1375 | 0.1512 | 1 | 0.1512 | 4.4169 | 0.0737 |
| AB | 0.0715 | 0.0205 | 1 | 0.0205 | 0.5980 | 0.4647 |
| A^2^ | -0.3018 | 0.6335 | 1 | 0.6335 | 18.5108 | 0.0036* |
| B^2^ | -0.2476 | 0.4265 | 1 | 0.4265 | 12.4620 | 0.0096* |
| Residual |  | 0.2396 | 7 | 0.0342 |  |  |
| Lack of Fit |  | 0.2347 | 3 | 0.0782 | 64.0432 | 0.0008* |
| Pure Error |  | 0.0049 | 4 | 0.0012 |  |  |
| Cor Total |  | 5.7552 | 12 |  |  |  |
| Std. Dev. | 0.1850 |  |  | R^2^ |  | 0.9584 |
| Mean | 4.1741 |  |  | Adjusted R^2^ |  | 0.9286 |
| C.V. % | 4.4319 |  |  | Predicted R^2^ | | 0.7087 |
| PRESS | 1.6764 |  |  | Adequate Precision | | 16.6971 |

* Significant difference at *p* < 0.05

**Table 3S** Regression of coefficients and ANOVA of the second-order model for enhancement of antioxidant activity of Miang extract by ultrasonic-assisted tannase treatment.

| I) ABTS radical scavenging activity | | | | | | |
| --- | --- | --- | --- | --- | --- | --- |
| Source | Coefficient | Sum of | df | Mean | *F*-Value | *p*-Value |
|  | Estimate | Squares |  | Square |  | Prob > *F* |
| Model | 2,475.6976 | 281622.50 | 9 | 31291.39 | 8.0608 | 0.0015* |
| A-Tannase | 76.2553 | 79412.82 | 1 | 79412.82 | 20.4571 | 0.0011* |
| B-Temp | 2.5187 | 86.64 | 1 | 86.64 | 0.0223 | 0.8842 |
| C-Time | 77.8685 | 82808.31 | 1 | 82808.31 | 21.3318 | 0.0010* |
| AB | 13.0446 | 1361.30 | 1 | 1361.30 | 0.3507 | 0.5669 |
| AC | -19.8758 | 3160.39 | 1 | 3160.39 | 0.8141 | 0.3881 |
| BC | 1.2929 | 13.37 | 1 | 13.37 | 0.0034 | 0.9544 |
| A^2^ | -45.0134 | 29200.26 | 1 | 29200.26 | 7.5221 | 0.0207* |
| B^2^ | -79.5416 | 91178.22 | 1 | 91178.22 | 23.4880 | 0.0007* |
| C^2^ | -26.8074 | 10356.45 | 1 | 10356.45 | 2.6679 | 0.1334 |
| Residual |  | 38819.13 | 10 | 3881.91 |  |  |
| Lack of Fit |  | 34886.62 | 5 | 6977.32 | 8.8713 | 0.0159* |
| Pure Error |  | 3932.51 | 5 | 786.50 |  |  |
| Cor Total |  | 320441.63 | 19 |  |  |  |
| Std. Dev. | 62.3050 |  |  | R^2^ | | 0.8789 |
| Mean | 2372.3410 |  |  | Adjusted R^2^ | | 0.7698 |
| C.V. % | 2.6263 |  |  | Predicted R^2^ | | 0.1545 |
| PRESS | 270926.0468 |  |  | Adequate Precision | | 8.9049 |
| II) DPPH radical scavenging activity | | | | | | |
| Source | Coefficient | Sum of | df | Mean | *F*-Value | *p*-Value |
|  | Estimate | Squares |  | Square | Value | Prob > *F* |
| Model | 1,744.2180 | 160366.30 | 9 | 17818.48 | 10.0027 | 0.0006* |
| A-Tannase | 32.4018 | 14337.97 | 1 | 14337.97 | 8.0488 | 0.0176* |
| B-Temp | 10.6422 | 1546.73 | 1 | 1546.73 | 0.8683 | 0.3734 |
| C-Time | 71.7059 | 70219.89 | 1 | 70219.89 | 39.4190 | < 0.0001* |
| AB | 8.1262 | 528.28 | 1 | 528.28 | 0.2966 | 0.5980 |
| AC | 16.7195 | 2236.34 | 1 | 2236.34 | 1.2554 | 0.2887 |
| BC | -2.3488 | 44.13 | 1 | 44.13 | 0.0248 | 0.8781 |
| A^2^ | -27.1253 | 10603.61 | 1 | 10603.61 | 5.9525 | 0.0349* |
| B^2^ | -64.3628 | 59699.69 | 1 | 59699.69 | 33.5133 | 0.0002* |
| C^2^ | -27.6162 | 10990.82 | 1 | 10990.82 | 6.1699 | 0.0323* |
| Residual |  | 17813.73 | 10 | 1781.37 |  |  |
| Lack of Fit |  | 14499.73 | 5 | 2899.95 | 4.3753 | 0.0655 |
| Pure Error |  | 3314.00 | 5 | 662.80 |  |  |
| Cor Total |  | 178180.03 | 19 |  |  |  |
| Std. Dev. | 42.2063 |  |  | R^2^ | | 0.9000 |
| Mean | 1662.8885 |  |  | Adjusted R^2^ | | 0.8100 |
| C.V. % | 2.5381 |  |  | Predicted R^2^ | | 0.3520 |
| PRESS | 115466.7835 |  |  | Adequate Precision | | 8.5055 |

* Significant difference at *p* < 0.05

**Supplementary Figure**

**Fig. 1S.** The example HPLC chromatograms of untreated and tannase treated Miang extracts.
